# Supplementary material for: Carbonylolysis of waste polyesters into high-value organic acids
Source: Nat Commun. 2026 Mar 10;17:2279. doi: 10.1038/s41467-026-70412-4 (PMC12976297; doi:10.1038/s41467-026-70412-4)
Supplement: Supplementary file 1 — Supplementary Information [file 41467_2026_70412_MOESM1_ESM.pdf]

Supplementary Information

Carbonylolysis of waste polyesters into high-value organic acids

Dongxu Liu [a, b], Siming Zhu [a, b], Qingqing Mei [a, b *]

[a]State Key Laboratory of Soil Pollution Control and Safety, Zhejiang University, Hangzhou, 310058, China.

[b]Institute of Environment Science and Technology, College of Environmental and Resource Sciences, Zhejiang University, Hangzhou, 310058, China.

*Corresponding author: Qingqing Mei

*E-mail: meiqq@zju.edu.cn

Address: 866 Yuhangtang Road, Hangzhou 310058, China.

Table of Contents

1. General Information 3

2. Exploration of reaction conditions 6

3. Study of reaction mechanism 14

4. Catalytic cycle 19

5. DFT calculation 25

6. LCA and TEA analysis 27

1. General Information

1.1 Materials:

Analytical grade solvents and commercially available reagents were purchased from commercial sources and used directly without further purification unless otherwise stated. Poly(ethylene terephthalate) (PET, Mn ~ 150,000, low-shear–pulverized PET powder without further ball milling) was purchased from Tesulang Chemical Materials Co., LTD., bis(2-hydroxyethyl) terephthalate (BHET, ≥ 93%, Innochem), ethylene glycol (EG, AR, Sinopharm Group Chemical reagent Co., LTD), carbon monoxide (CO, Hangzhou Jinggong Co. LTD.), hexafluoroisopropanol (HFIP, 99.5% , Aladdin), toluene (TL, AR, Sinopharm Group Chemical reagent Co., LTD), acetonitrile (ACN, AR, Sinopharm Group Chemical reagent Co., LTD), cyclohexane (CYH, AR, Sinopharm Group Chemical reagent Co., LTD), acetic acid (HAc, GR, Sinopharm Group Chemical reagent Co., LTD), dimethyl isosorbide (DMI, ≥ 98%, Shanghai Adamas Reagent Co., LTD), γ-valerolactone (GVL, ≥ 98.0%, Shanghai Aladdin Biochemical Technology Co., LTD), distilled water. Catalysts include rhodium trichloride ($RhCl_3 \cdot 3H_2O$, 99%, Innochem), iridium trichloride ($IrCl_3 \cdot 3H_2O$, Innochem), palladium chloride ($PbCl_2$, 99.9%, Innochem), ruthenium chloride ($RuCl_3$, 99.5%, Innochem), cobalt chloride ($CoCl_2 \cdot 6H_2O$, 99.99%, Rhawn) and nickel chloride ($NiCl_2 \cdot 6H_2O$, Shandong Xiya Chemical Technology Co. LTD.), the above catalysts were combined with distilled water added in the reaction to form an aqueous solution of appropriate concentration. Catalytic additives include lithium iodide (LiI, 99%, Alfa), zinc iodide (ZnI_2, ≥ 99.0%, Shanghai D&B Biological Science and Technology Co., LTD), iodomethane (CH_3I, 99%, Aladdin), cuprous iodide (CuI_2, 99%, Shanghai Meryer Chemical Technology Co., LTD) sodium iodide (NaI, 99.5%, Rhawn), iodoethane (C_2H_5I, 99%, Energy Chemical Co., LTD), 1,2-diiodoethane ($C_2H_4I_2$, 98%, Shanghai D&B Biological Science and Technology Co., LTD), 2-iodoethanol (C_2H_5IO, 99.7%, Shanghai Bide Pharmatech Co., LTD) and hydriodic acid (HI, 55-57%, Shanghai Titan Scientific Co., LTD). Other reagents include Propionic acid (PA, AR, Sinopharm Group Chemical reagent Co., LTD), butyric acid ($C_4H_8O_2$, AR, Sinopharm Group Chemical reagent Co., LTD), valeric acid ($C_5H_{10}O_2$, CP, Sinopharm Group Chemical reagent Co., LTD), glutaric acid (GA, 99%, Shanghai Macklin Biochemical Co., LTD), adipic acid (AA, AR, Aladdin), mesitylene (≥ 98%, Aladdin), chloroethane (Dalian keriqiti Co., LTD), fumaric acid (FA, 99%, Aladdin) sodium hydroxide (NaOH, AR, ≥ 92.0%, Sinopharm Group Chemical reagent Co., LTD), Dimethyl sulfoxide-d_6 (DMSO, 99.8%, Energy Chemical) and deuterium oxide (D_2O, 99%, Innochem). The polymers are polyethylene 2,5-furandicarboxylate (PEF, Alfa), polyethylene glycol succinate (PES, 98%, Bide Pharmatech LTD.), polyethylene adipate (PEA, Mw: ~1000, Aladdin), polyethylene glycol (PEG, Macklin), polybutylene terephthalate (PBT, Alfa) and polytrimethylene terephthalate (PTT, Mitsubishi Group),

polybutylene adipate-co-terephthalate (PBAT, GlpBio Technology), Bromine (Br_2, Shanghai Lingfeng Chemical Reagent Co. LTD.), Sodium thiosulfate pentahydrate ($Na_2S_2O_3 \cdot 5H_2O$, Sinopharm Group Chemical reagent Co., LTD), Sulfuric acid (H_2SO_4, AR, Sinopharm Group Chemical reagent Co., LTD), Starch indicator (1% w/v in water, Aladdin).

1.2 Reactor equipment:

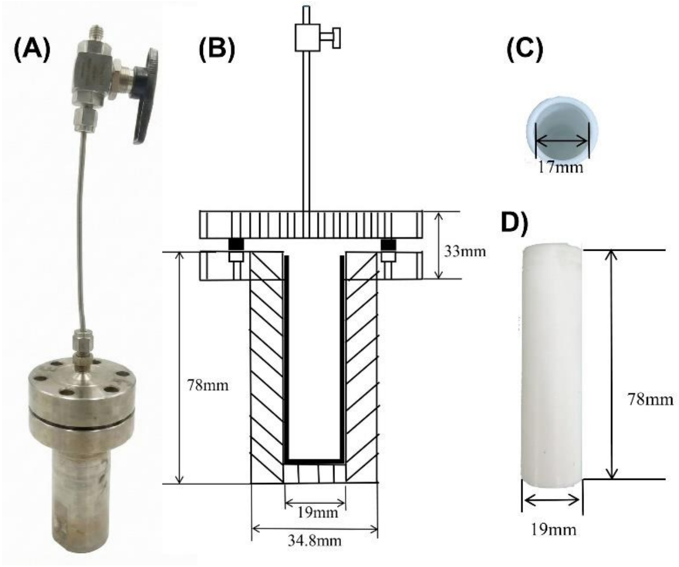

Fig. S1. The reaction apparatus. (A) Photograph of the high-pressure reactor; (B) Cutaway view of the high-pressure reactor; (C) Cross-sectional view of the reactor liner; (D) Side view of the reactor liner. All carbonylolysis reactions were conducted in a stainless-steel stirred autoclave manufactured by Ruisheng Laboratory Equipment Factory (Lyushunkou District, Dalian). The reactor is a high-pressure-resistant steel vessel equipped with a PTFE liner. The total internal volume of the autoclave was 24 mL, and the usable volume of the PTFE liner was 19.4 mL.

2. Exploration of reaction conditions

2.1 Catalytic ion:

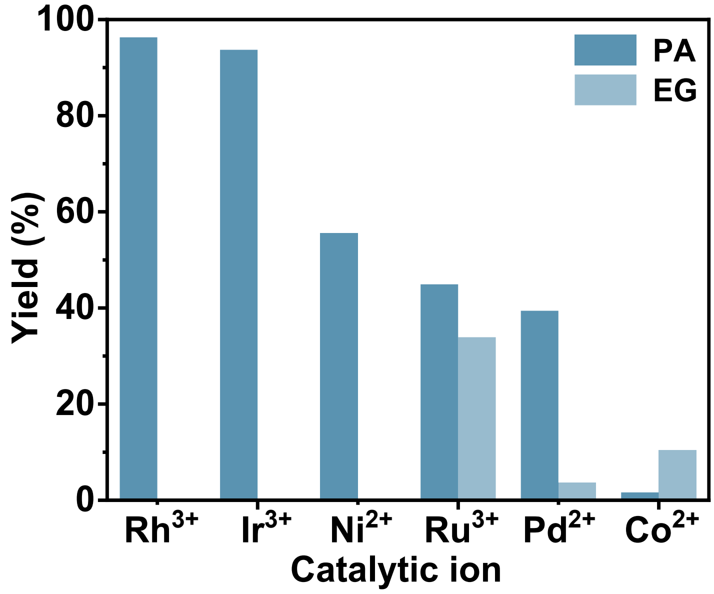

Fig. S2. Effect of catalyst on PET carbonylolysis. Reaction conditions: PET (0.192 g, 1 mmol structural unit), HFIP (2 mL), H_2O (0.5 mL), CH_3I (0.5 mmol), CO (2 MPa) and catalyst (5 mol%) at 170°C for 12 h.

2.2 Iodide additive:

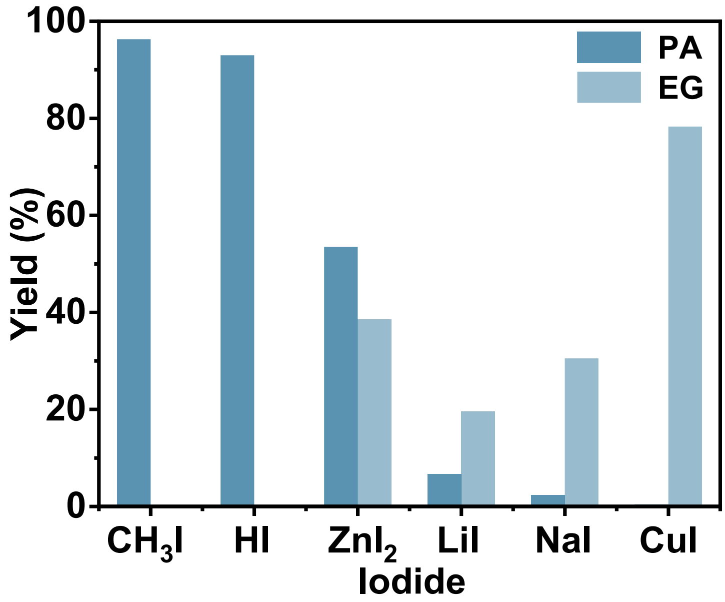

Fig. S3. Effect of iodide additive on PET carbonylolysis. Reaction conditions: PET (0.192 g, 1 mmol structural unit), HFIP (2 mL), H_2O (0.5 mL), iodide additive (0.5 mmol, except ZnI_2 0.25 mmol), CO (2 MPa) and catalyst ($RhCl_3$, 5 mol%) at 170°C for 12 h.

2.3 Solvent:

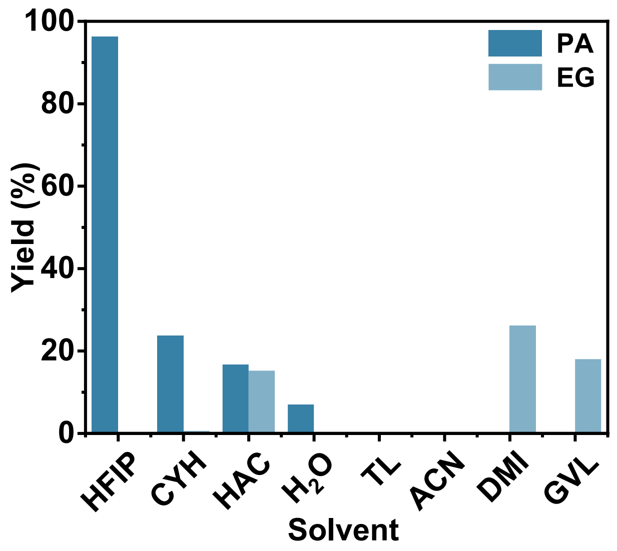

Fig. S4. Effect of solvent on PET carbonylolysis. Reaction conditions: PET (0.192 g, 1 mmol structural unit), solvent (2 mL), H_2O (0.5 mL), CH_3I (0.5 mmol), CO (2 MPa) and catalyst ($RhCl_3$, 5 mol%) at 170°C for 12 h. HFIP: hexafluoroisopropanol; CYH: cyclohexane; HAC: acetic acid; TL: toluene; ACN: acetonitrile; DMI: dimethyl isosorbide; GVL: γ-valerolactone.

2.4 Rh^{3+} amount:

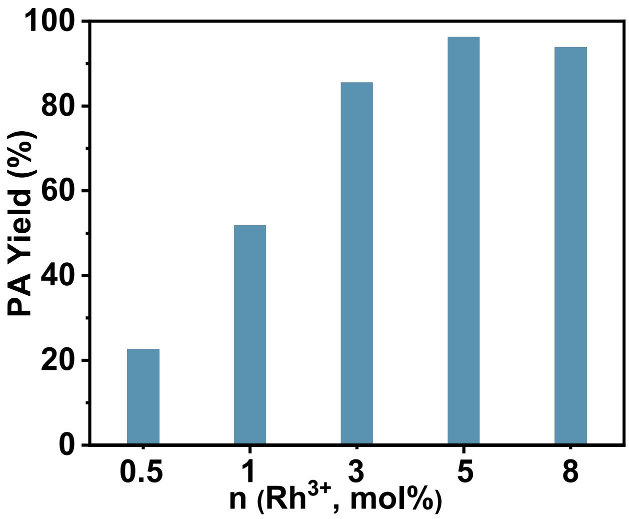

Fig. S5. Effect of Rh^{3+} amount on PET carbonylolysis. Reaction conditions: PET (0.192 g, 1 mmol structural unit), HFIP (2 mL), H_2O (0.5 mL), CH_3I (0.5 mmol), CO (2 MPa) and $RhCl_3$ at 170°C for 12 h.

2.5 Counter ions

Table S1. Carbonylolysis performance of different anionic rhodium salts.

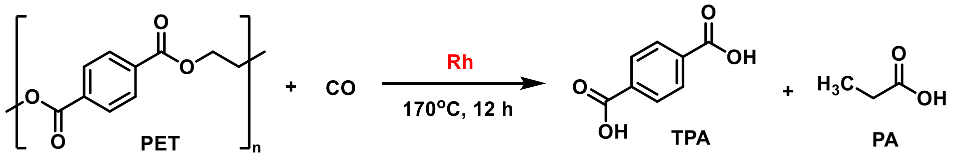

No.	Rh	HI	TPA Yield (%)	PA Yield (%)	EG Yield (%)
1	RhI_3	HI	>99	97.6	0.0
2	RhI_3	-	97.4	13.0	83.8
3	$RhCl_3$	HI	>99	93.0	0.0
4	$RhCl_3$	-	93.6	0.1	76.7

Standard reaction conditions: PET (0.192g, 1 mmol structural unit), HFIP (2 mL), H_2O (0.5 mL), HI (0.4 mmol), CO (2 MPa) and catalyst ($RhCl_3$ or RhI_3, 5 mol%) at 170°C for 12 h.

2.6 CH_3I content:

Table S2. Reaction performance of different CH_3I content.

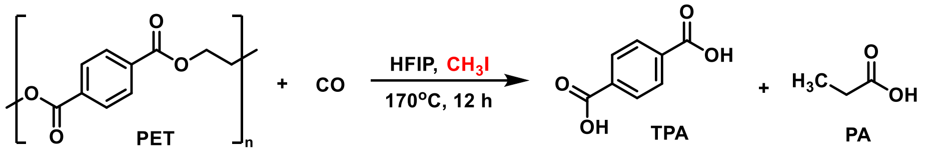

No.	CH_3I loading	TPA Yield (%)	PA Yield (%)
1	0.10	95.1	0.33
2	0.12	96.2	5.0
3	0.15	96.8	6.8
4	0.18	97.3	17.3
5	0.20	99.0	83.0
6	0.30	97.5	85.4

Standard reaction conditions: PET (0.192g, 1 mmol structural unit), solvent (2 mL), H_2O (0.5 mL), CH_3I (0.5 mmol), CO (2 MPa) and catalyst ($RhCl_3$, 5 mol%) at 170°C for 12 h.

2.7 GC Quantification of Product Yields

Quantitative analysis of PA and EG in the liquid phase is performed using Shimadzu GC-2014 gas chromatography equipped with Shimadzu SH-WAX Polyethylene glycol GC Column. The yields of PA and EG were calculated from gas chromatography chromatograms using mesitylene as the internal standard. The composition of the reaction mixture was determined by equation E1.

$$N_i = K_i \times \frac{S_i}{S_s} \times N_s \times 100\% \qquad \text{(E1)}$$

Where N_i is the molar amount of component i, K_i is the response of component i, and S_i is the peak area of component i. N_s is the molar amount of the internal standard mesitylene and S_s is the peak area of internal standard mesitylene. Figure S6-I presents the GC calibration curves for PA and EG using mesitylene as the internal standard, from which the corresponding response factors (K_i) were obtained.

The yield of PA and EG are defined as the ratio of the actual moles of PA and EG formed to the theoretical moles of PA and EG, respectively (equation E2, E3).

$$PA\ Yield\ (\%) = \frac{PA\ amount\ quantified\ by\ GC\ (mmol)}{Theoretical\ PA\ amount\ (mmol)} \times 100\% \qquad \text{(E2)}$$

$$EG\ Yield\ (\%) = \frac{EG\ amount\ quantified\ by\ GC\ (mmol)}{Theoretical\ EG\ amount\ (mmol)} \times 100\% \qquad \text{(E3)}$$

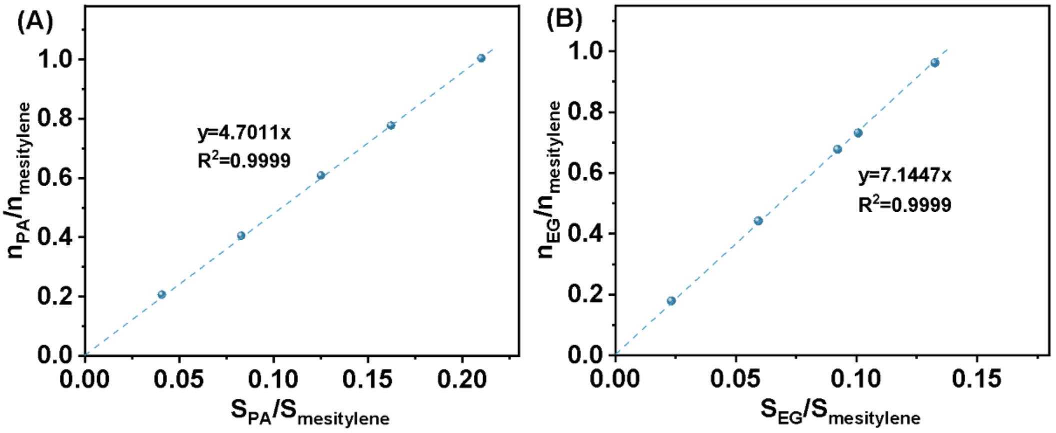

Fig. S6-I. Gas chromatography calibration curves for (A) propionic acid (PA) and (B) ethylene glycol (EG), obtained using mesitylene as the internal standard.

As representative cases, GC chromatograms obtained in the presence and absence of an iodide promoter are shown. Under the optimized conditions (170°C, CO pressure of 2 MPa, and a reaction time of 12 h), using $RhCl_3$ as the catalyst and CH_3I as the promoter, PET was nearly completely converted to PA. The corresponding GC chromatogram (Fig. S6-II) exhibits four peaks assigned to mesitylene (internal standard), HFIP (solvent), acetic acid (by-product), and PA (product). Based on Equations E1 and E2, a PA yield of 96% was obtained. In contrast, in the absence of an iodide promoter, the GC chromatogram (Fig. S6-III) shows

peaks corresponding to mesitylene, HFIP, acetic acid, EG, and only trace amounts of PA, yielding EG as the predominant product (EG yield = 76.7%).

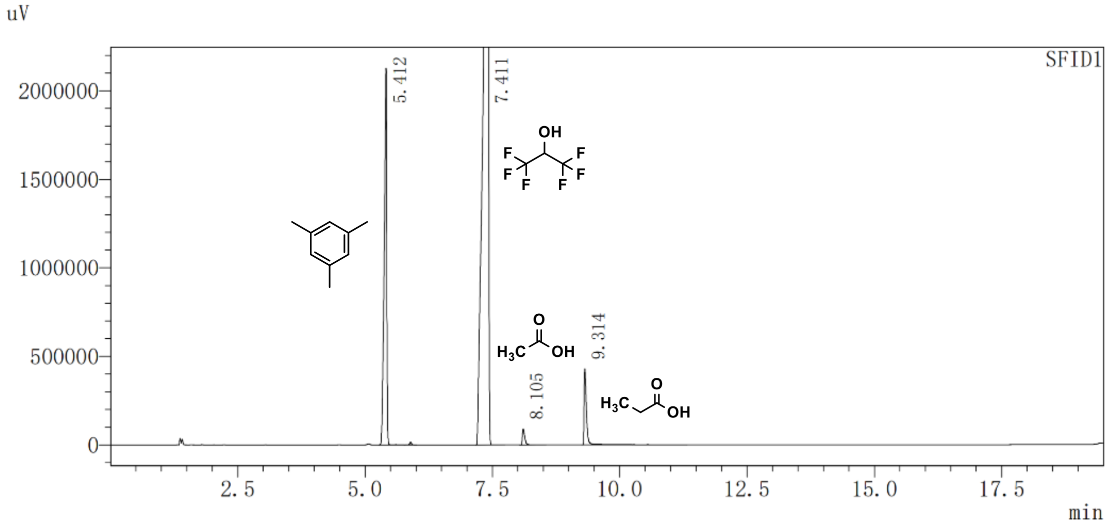

Fig. S6-II. GC chromatogram of the reaction mixture obtained under the optimized conditions.

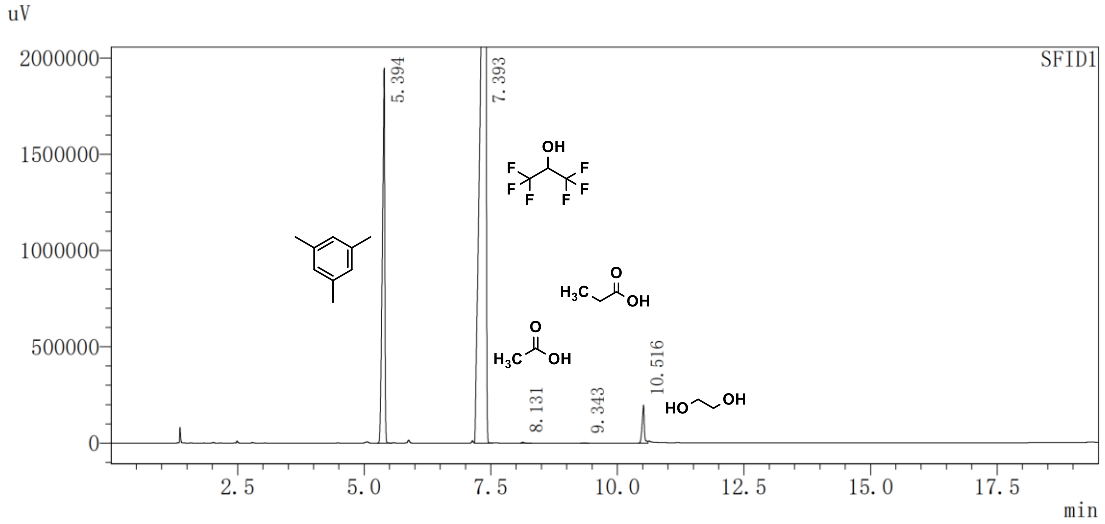

Fig. S6-III. GC chromatogram of the reaction mixture obtained under identical conditions in the absence of an iodide promoter.

2.8 Particle-size–dependent experiment

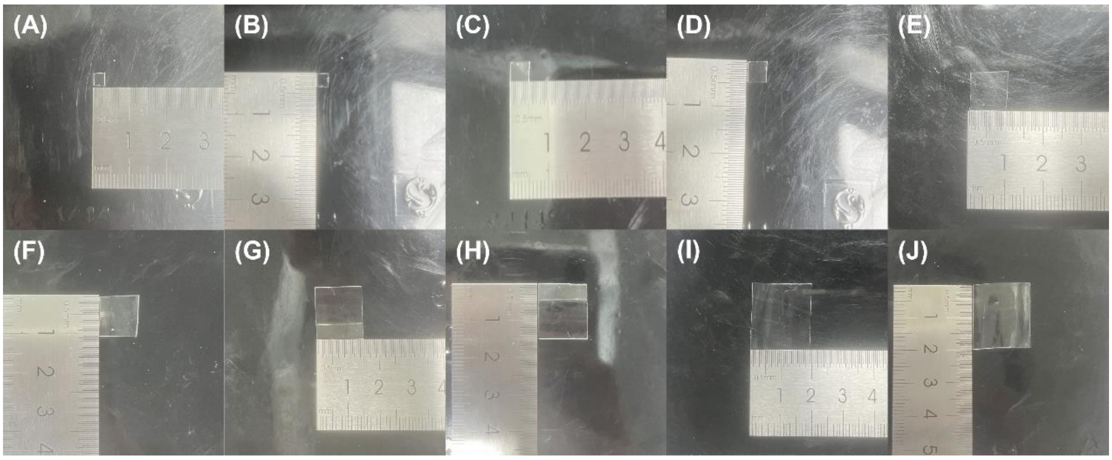

Fig. S7. Different sizes of PET bottle flakes. (A-B) 0.3 × 0.3 cm^2; (C-D) 0.5 × 0.5 cm^2; (E-F) 1.0 × 1.0 cm^2; (G-H) 1.5 × 1.5 cm^2; (I-J) 2.0 × 2.0 cm^2.

Table S3. Reaction performance of different PET samples sizes.

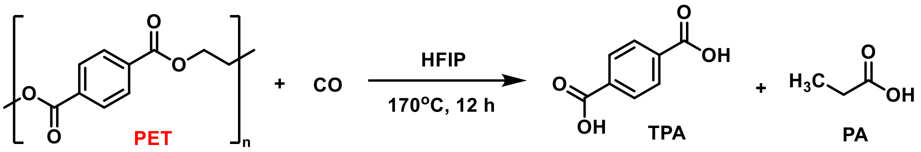

No.	PET samples size	TPA Yield (%)	PA Yield (%)
1	2.0 × 2.0	94.6	93.6
2	1.5 × 1.5	91.2	90.6
3	1.0 × 1.0	95.1	92.5
4	0.5 × 0.5	92.7	91.6
5	0.3 × 0.3	96.0	92.2
6	randomly irregular mixture	94.1	92.1

Standard reaction conditions: PET (0.192g, 1 mmol structural unit), solvent (2 mL), H_2O (0.5 mL), CH_3I (0.5 mmol), CO (2 MPa) and catalyst ($RhCl_3$, 5 mol%) at 170°C for 12 h.

3. Study of reaction mechanism

3.1 ^{19}F-NMR of HFIP under different reaction conditions:

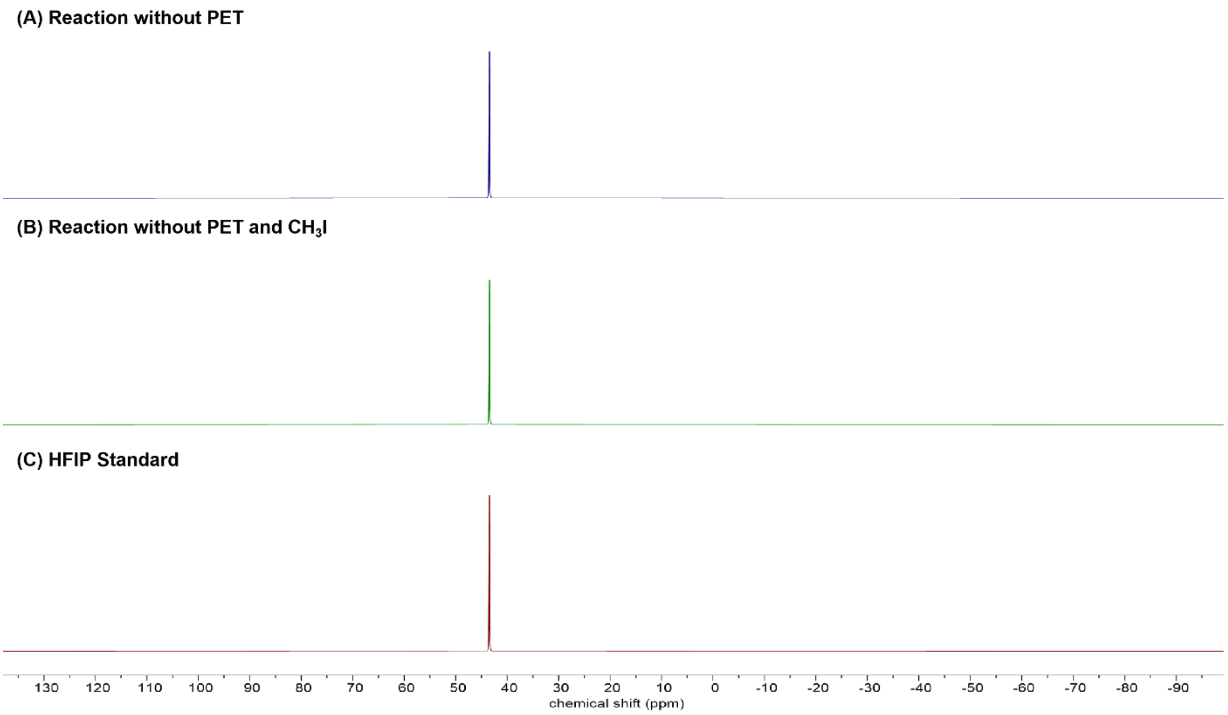

Fig. S8. Evaluation of HFIP stability under catalytic conditions by ^{19}F-NMR (D_2O). (A) Reaction solutions without PET: HFIP (2 mL), H_2O (500 μL), CH_3I (0.5 mmol), CO (2 MPa) and catalyst ($RhCl_3$, 5mol%) at 170°C for 12 h; (B) Reaction solutions without PET and CH_3I: HFIP (2 mL), H_2O (500 μL), CO (2 MPa) and catalyst ($RhCl_3$, 5mol%) at 170°C for 12 h. (C) HFIP standard. These spectra were acquired with appropriate signal suppression to ensure that minor fluorinated species, if present, would be observable. ^{19}F NMR spectra analysis showed no detectable changes in the dominant HFIP resonance and no additional fluorine-containing signals, indicating that HFIP remains chemically stable in the presence of Rh and CO.

3.2 Exploration of the overall reaction pathway:

Table S4. Carbonylation of EG and carbonylolysis of BHET and PET.

No.	Reactant (1 mmol)	PA Yield (%)	EG Yield (%)
1	EG	98.3	0.4
2	BHET	98.8	0.0
3	PET	96.3	0.2

Reaction conditions: reactant (1 mmol), HFIP (2 mL), H_2O (500 μL), CH_3I (0.5 mmol), CO (2 MPa) and catalyst ($RhCl_3$, 5mol%) at 170°C for 12 h.

3.3 Exploration of reaction intermediates:

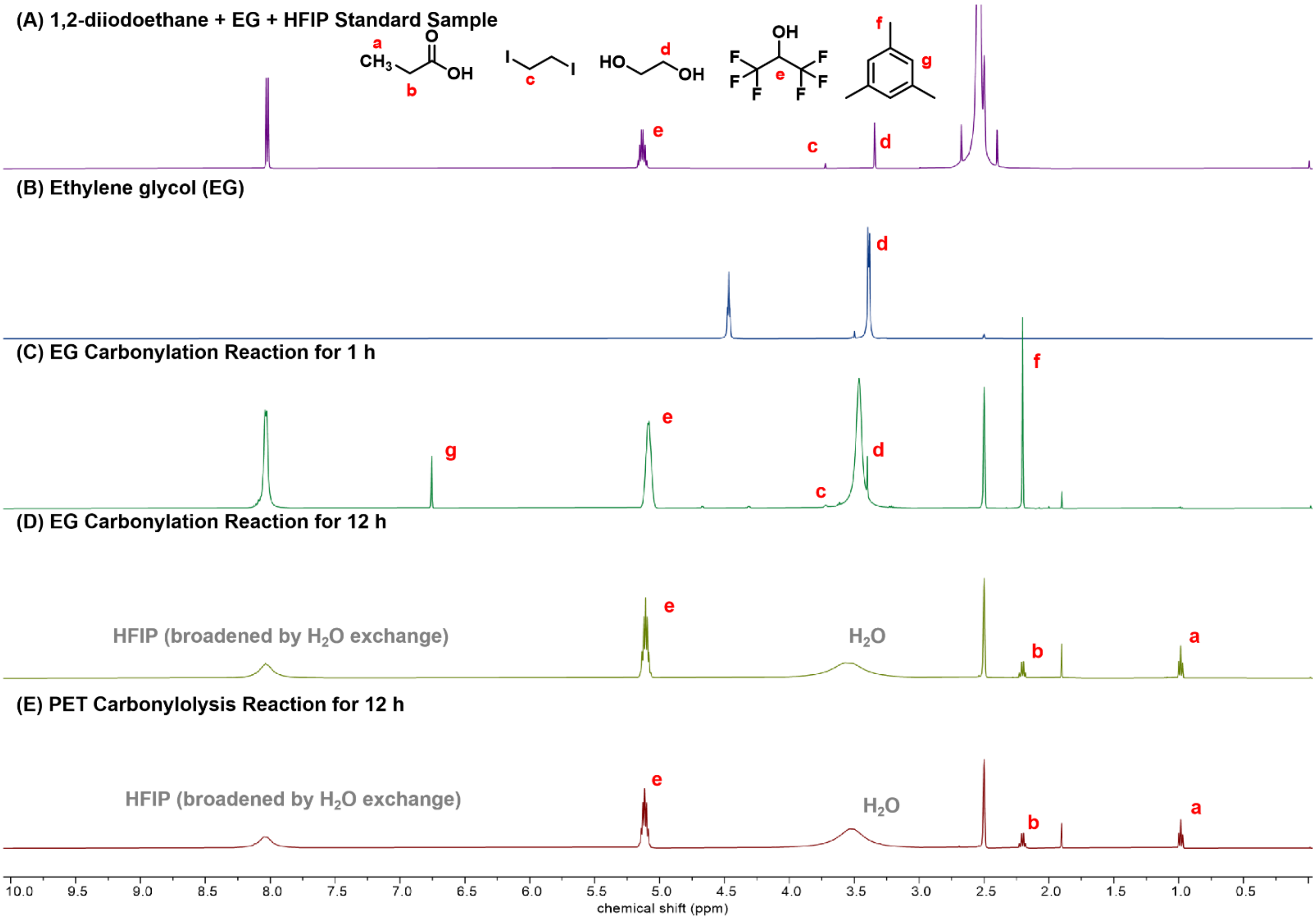

Fig. S9. ^1H NMR (DMSO-d$_6$) spectra of (A) a mixed standard sample containing 1,2-diiodoethane, EG, and HFIP; (B) EG standard; (C) reaction mixture obtained after 1 h of EG carbonylation; (D) reaction mixture obtained after 12 h of EG carbonylation; and (E) reaction mixture obtained after 12 h of PET carbonylolysis. Reaction conditions: EG (1 mmol) or PET (0.192 g, 1 mmol of structural units), HFIP (2 mL), H_2O (0.5 mL), CO (2 MPa), $RhCl_3$ (5 mol%), 170°C.

3.4 Exploration of reaction products:

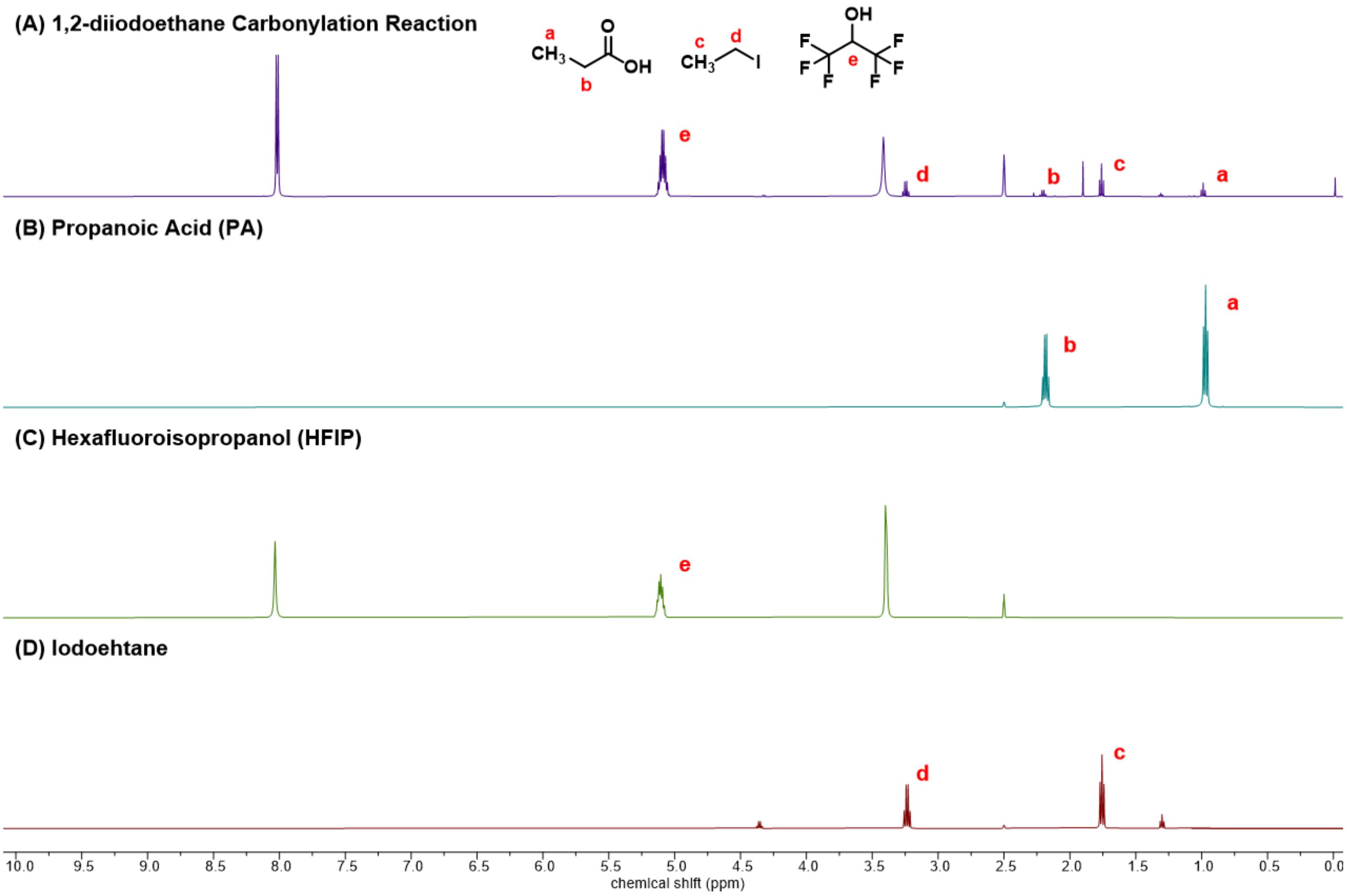

Fig. S10. ^{1}H-NMR (DMSO-d$_6$) of (A) reaction mixture for 1,2-diiodoethane carbonylation without additional iodine. Reaction conditions: 1,2-diiodoethane (1 mmol), HFIP (2 mL), H_2O (0.5 mL), CO (2 MPa) and catalyst ($RhCl_3$, 5mol%) at 170°C for 12 h. (B) PA standard; (C) HFIP standard; (D) Iodoethane standard.

4. Catalytic cycle

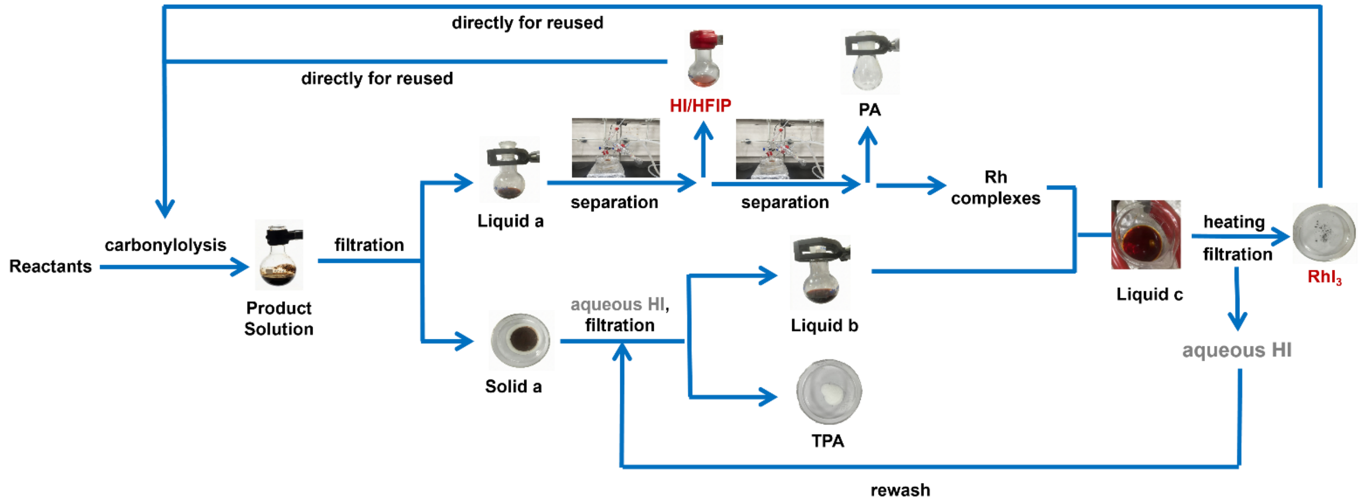

Fig. S11. The cyclic process of the PET carbonylolysis reaction within the laboratory setting.

Due to equipment limitations, laboratory-scale recycling experiments cannot fully replicate the high-pressure filtration or centrifugal separation used in industrial processes. Consequently, a small fraction of Rh species may precipitate following CO release and become entrained in the solid TPA phase. To ensure complete material balance under laboratory conditions, an additional aqueous HI extraction step was deliberately employed to recover trace Rh from the TPA solid. Laboratory PET carbonylolysis reaction system recycling procedure: After completion of the reaction, the crude mixture was centrifuged to separate the solid fraction (*Solid a*) from the liquid phase (*Liquid a*). *Solid* a was washed with aqueous HI to dissolve any trace Rh precipitate that might be present, and the resulting suspension was centrifuged. The recovered solid was then recrystallized to afford purified TPA, and the wash solution was collected as *Liquid b*. *Liquid a* was distilled to recover the HI/HFIP mixture, which was directly reused in the repeated catalytic cycle. The remaining liquid was evaporated to recover PA. The final residue, containing Rh complexes, was combined with *Liquid b* (denoted as *Liquid c*) and thermally aged to promote precipitation of RhI_3. The recovered aqueous HI solution could be reused in the subsequent TPA washing step. It is worth noting that this aqueous HI is specifically reserved for the TPA purification cycle and does not enter the catalytic reaction. The resulting non-volatile Rh-containing residue including RhI_3, together with the recovered HI/HFIP mixture, was directly reused in subsequent repeated catalytic cycle without the addition of extra iodide. Using this procedure, the recycled catalytic system exhibited comparable activity and selectivity to the fresh system.

4.1 Rh Recovery and Reactivation

Table S5. Reaction performance of Rh from different sources.

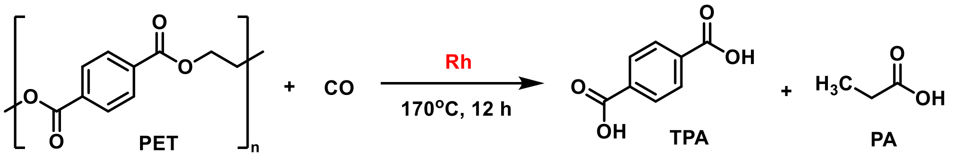

No.	Rh	I^-	TPA Yield (%)	PA Yield (%)	EG Yield (%)
1	$RhCl_3$	CH_3I	>99	96.0	0.0
2	RhI_3	HI	>99	97.6	0.0
3	Rh-cyclic	HI	>99	95.9	0.0

Standard reaction conditions: PET (0.192g, 1 mmol structural unit), HFIP (2 mL), H_2O (0.5 mL), CH_3I (0.5 mmol) or HI (0.4 mmol), CO (2 MPa) and catalyst (Rh from different sources, 5 mol%) at 170°C for 12 h.

Rh was recovered following the above procedure and isolated as RhI_3. Under standard reaction conditions, the recycled Rh catalyst (Rh-cyclic) exhibited activity and selectivity comparable to fresh $RhCl_3$ and simulated recycled RhI_3 (prepared by thermal aging of $RhCl_3$ in excess aqueous HI) when used with fresh HFIP and HI, confirming efficient reactivation and preservation of catalytic integrity (Table S4).

Reactivation pathway of RhI_3: Upon re-exposure to CO and HI under the reaction conditions, isolated RhI_3 is converted in situ into catalytically active Rh–iodide carbonyl species ($H_zRh(CO)_xI_y$)[1-3]. This conversion occurs via coordination of CO and HI to Rh, which restores the Rh center to its homogeneous, catalytically competent state. The resulting Rh–iodide carbonyl complexes are well-known active species in liquid-phase carbonylation reactions, consistent with established Rh carbonyl chemistry. This reactivation ensures that the recycled Rh catalyst retains comparable activity and selectivity to fresh $RhCl_3$.

It is important to note that conversion of Rh species to RhI_3 is not required for catalyst reuse under the studied reaction conditions. During catalysis, Rh remains fully dissolved in the homogeneous reaction mixture and retains full catalytic activity. Formation of RhI_3 occurs only after removal of CO and volatile solvent components and therefore does not represent the operative catalytic species. In fact, after reaction completion, solvent recovery, and product isolation, the majority of Rh remains in the non-volatile residue, which could be directly reused. ICP–MS analysis of the residue indicates near-quantitative Rh retention (~99%, within analytical error) under these laboratory conditions, demonstrating that the majority of Rh remains in the non-volatile catalytic phase and supporting its effective recyclability. Direct reuse of this Rh-containing residue in subsequent cycles, without deliberate precipitation of RhI_3, delivers 97% PA yield,

consistent with fresh runs. This result demonstrates effective recyclability and catalytic stability under closed-system operation. Although industrial-scale operation was not evaluated, this behavior is conceptually consistent with established industrial practice in liquid-phase Rh-catalyzed carbonylation, where Rh is maintained in a high-boiling catalytic phase and recovered primarily through product distillation[2-4].

Therefore, isolation of Rh as RhI_3 was employed solely as a laboratory-scale, quantitative method to account for the total Rh content, representing an extreme case of the complex mixture of Rh species in the catalytic residue rather than an intrinsic or necessary feature of the catalytic operation. In this procedure, all Rh is deliberately isolated for mass-balance analysis rather than retained in the catalytic phase. As ICP–MS analysis of the residue indicates near-quantitative Rh retention, using RhI_3 as a representative species does not imply actual Rh loss in the TEA. This conservative treatment does not affect the qualitative conclusions regarding the robustness and recyclability of the catalytic system.

4.2 HFIP cycling

Following the recycling procedure described above (Figures S10), HFIP was recovered together with HI as the volatile fraction (HI/HFIP) after each run with near-quantitative efficiency (>99%) and reused for at least three consecutive reaction cycles. Because HFIP and HI function cooperatively as the reaction medium, separation of HI from HFIP is neither required nor desirable for effective solvent circulation. Moreover, co-recycling of the solvent and iodide promoter is consistent with established industrial practice in Monsanto/Cativa-type carbonylation processes. ^{1}H, ^{13}C and ^{19}F NMR analyses of the recycled solvent showed no detectable impurity accumulation relative to fresh HFIP. Consistently, PET conversion and product selectivity remained essentially unchanged over multiple cycles with fresh $RhCl_3$, indicating that solvent purity and catalytic performance were preserved upon reuse (Fig. S12–S14, Table S6). These results experimentally demonstrate that HFIP can be efficiently recovered and reused without loss of solvent purity or catalytic performance, validating the feasibility of circulating the reaction medium.

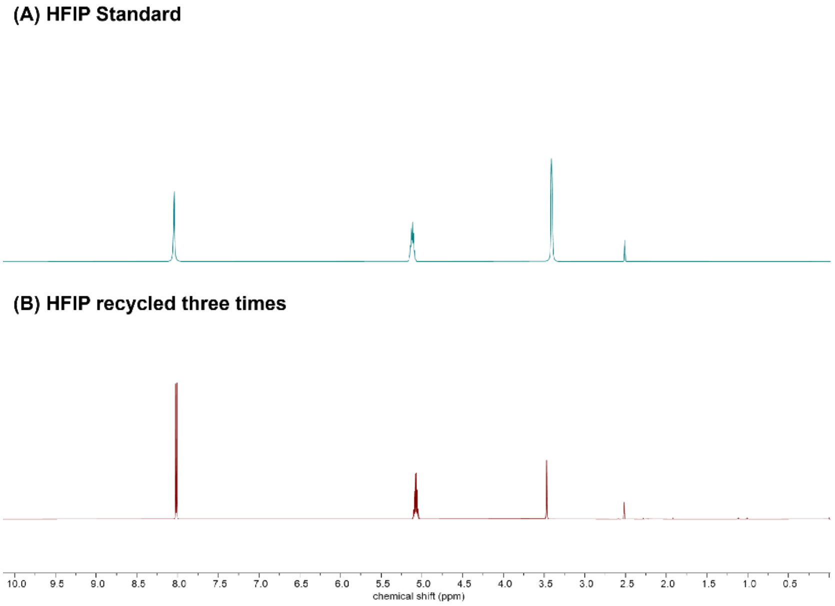

Fig. S12. ^{1}H-NMR (DMSO-d_6) of (A) HFIP standard; (B) HFIP recycled three times.

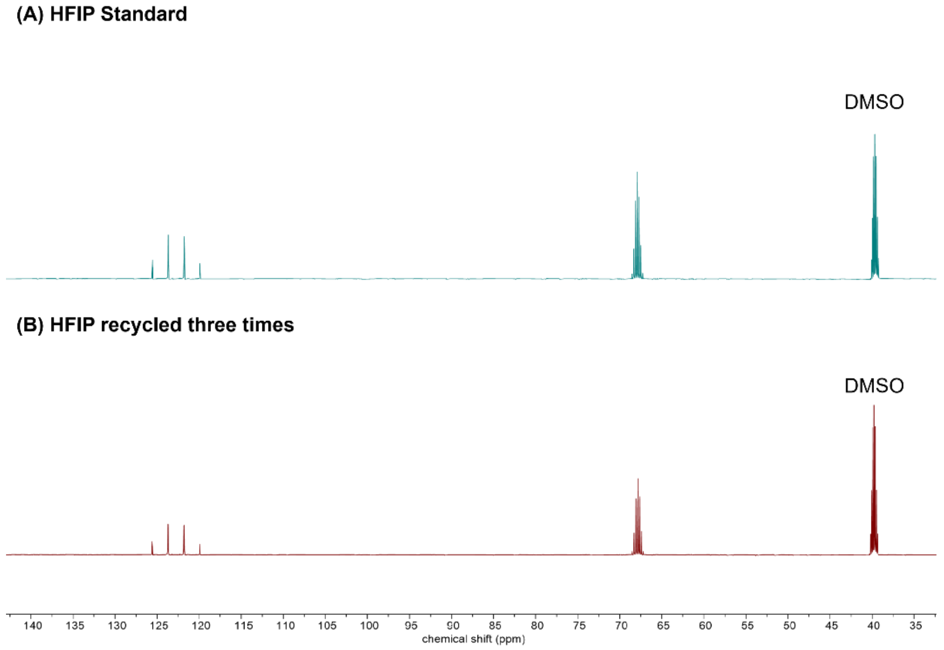

Fig. S13. ^{13}C-NMR (DMSO-d_6) of (A) HFIP standard; (B) HFIP recycled three times.

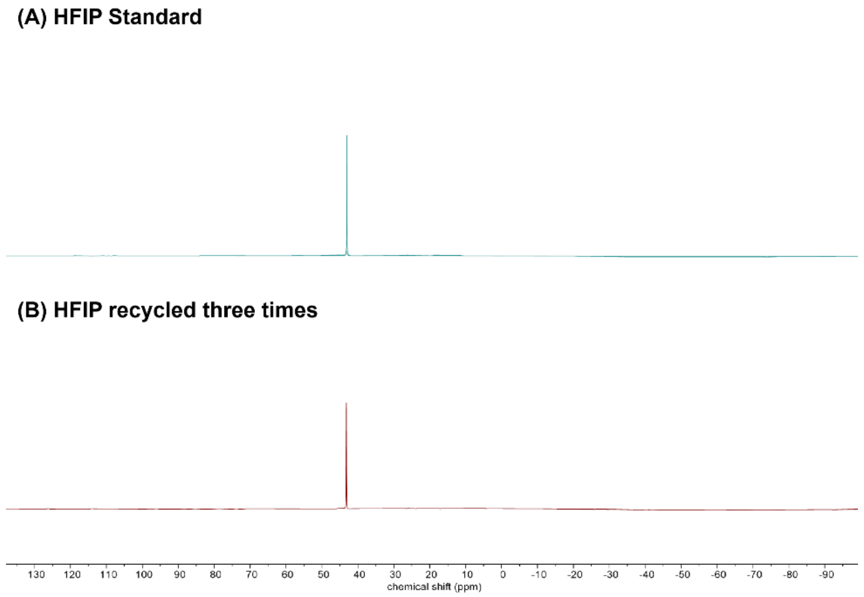

Fig. S14. ^{19}F-NMR (D_2O) of (A) HFIP standard; (B) HFIP recycled three times.

Table S6. Recyclability Test of HFIP.

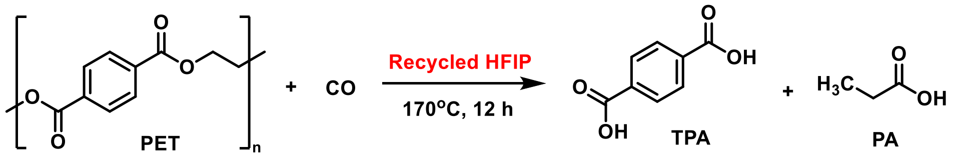

Cycle Number	Temp.	Sol.	TPA Yield (%)	PA Yield (%)
1	170°C	HFIP	99	96.3
2	170°C	HFIP	99	96.7
3	170°C	HFIP	99	96.4

Standard reaction conditions: PET (0.192g, 1 mmol structural unit), HFIP (2 mL), H_2O (0.5 mL), CH_3I (0.5 mmol), CO (2 MPa) and catalyst ($RhCl_3$, 5 mol%) at 170°C for 12 h.

4.3 Iodide reagent cycling

In this system, iodide is distributed between two fractions after the reaction. The major fraction is present as HI, which is dissolved in HFIP and remains volatile, while the remainder is retained within the non-volatile Rh-containing catalytic complex. Consequently, iodide recycling is intrinsically integrated with the recycling of HFIP and the Rh catalyst.

The volatile HI fraction was efficiently recovered together with HFIP by distillation and directly reused in subsequent runs. After solvent recovery and product isolation, the remaining non-volatile residue constituted the Rh-containing catalytic phase[1]. This phase, together with Rh species recovered as RhI_3 and the recovered HI/HFIP mixture, was directly reused in subsequent repeated cycle without the addition of extra iodide, affording a TPA yield of 98% and a PA yield of 90.9%. This procedure demonstrates the effective co-recycling of Rh, iodide, and HFIP within an integrated recycling scheme.

Under these non-closed laboratory operating conditions, the overall iodide recovery is determined to exceed 93%. It should be emphasized that this value represents a conservative estimate at the laboratory scale, rather than intrinsic losses expected under practical closed-system operation. Although minor iodide loss is unavoidable under laboratory-scale, non-closed recycling conditions, these results demonstrate that the iodide additive is largely retained and can be efficiently recycled within the present system. While industrial-scale operation was not examined, this behavior is conceptually consistent with established liquid-phase Rh/iodide carbonylation processes [5-7].

4.4 Determination of iodide content[8]

The distilled product solution was acidified to a slightly acidic pH with 10 mL of 10% dilute sulfuric acid, after which an excess of bromine water (Br_2) was added to quantitatively oxidize I^- toiodate (IO_3^-). Formic acid was then added dropwise with gentle shaking until the brown-red color of Br_2 completely disappeared. Subsequently, 5.0 g of potassium iodide (KI) was introduced into the acidic IO_3^- containing solution, which immediately turned brown due to the formation of elemental iodine (I_2). After standing for 5 minutes, the generated iodine was titrated with 0.2 mol/L sodium thiosulfate ($Na_2S_2O_3$) solution. When the solution color faded to pale yellow, 2 mL of starch indicator was added, and titration was conducted slowly until the blue color disappeared completely. The total iodine content in the original sample was then calculated from the concentration and consumption volume of the sodium thiosulfate solution.

In a typical run with 0.5 mmol of CH_3I, the iodine content in the distilled product solution after reaction was measured as 0.32 mmol. Taking into account the Rh retention determined by ICP–MS (0.05 mmol, 99%), the overall iodide recovery in the system was calculated to be (0.32 + 0.05 × 3 × 99%)/0.5=93%.

$$\mathrm{HI+Br_2 \rightarrow IBr+HBr}$$

$$\mathrm{IBr + 3H_2O + 2Br_2 \rightarrow HIO_3 + 5HBr}$$

$$\mathrm{HIO_3 + 5KI + 5H^+ \rightarrow 3I_2 + 3H_2O + 5K^+}$$

$$\mathrm{I_2 + 2Na_2S_2O_3 \rightarrow 2NaI + Na_2S_4O_6}$$

5. DFT calculation

5.1 Gibbs Free Energy calculation:

Table S7. Gibbs Free Energy for hydrolysis or carbonylolysis of PET.

No.	Decomposition Method	Gibbs Free Energy (kcal/mol)
1	Hydrolysis	2.43
2	Carbonylolysis	-82.34

6. LCA and TEA analysis

6.1 Comparison of iodide types:

Table S8. Reaction performance of HI substituted for CH_3I.

No.	Iodide	PA Yield (%)	TPA Yield (%)
1	CH_3I	96.3	> 99.0
2	HI	93.0	> 99.0

Reaction conditions: PET (1 mmol), HFIP (2 mL), H_2O (500 μL), CH_3I (0.5 mmol) or HI (0.4 mmol), CO (2 MPa) and catalyst ($RhCl_3$, 5mol%) at 170°C for 12 h.

6.2 Process flow design:

Supplementary Note 2: Process details and descriptions for recycling waste PET to obtain TPA and propionic acid via PET carbonylolysis system in ASPEN.

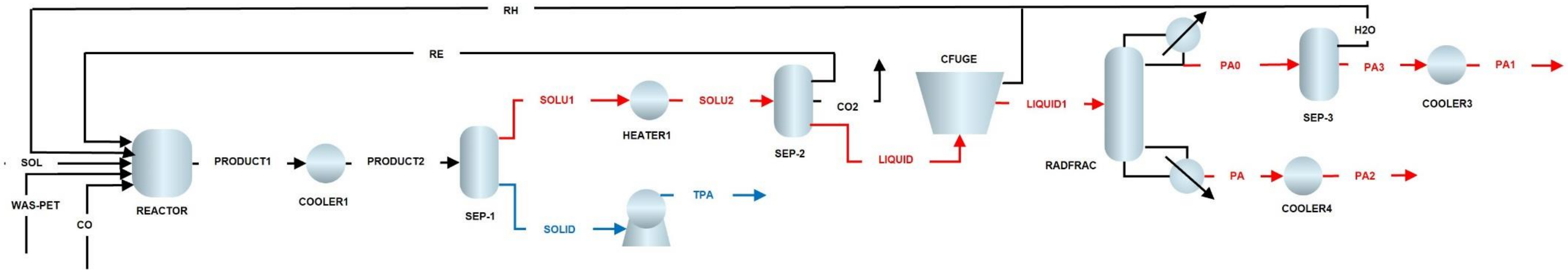

Figure S15. Process flow diagram for waste PET carbonylolysis to TPA and PA, simulated using Aspen Plus (PA stream shown in red and TPA stream shown in blue).

6.3 Mass balance:

Table S9-I. Mass balance of PET carbonylolysis system.

Para.	WAS-PET	SOL	CO	PRODUCT1	PRODUCT2	SOLID	TPA	SOLU1	SOLU2	RE
Temp./°C	25	25	25	170	33	33	33	33	33	60
Pres./bar	1.0	1.0	20.0	30.7	30.7	30.7	1.0	30.7	1.0	1.0
WATER	0	2400	0	56.3	56.3	0	0	56.3	56.3	0
CO	0	0	0	10702.5	10702.5	0	0	10702.5	10702.5	10702.5
PET	12500	0	0	0	0	0	0	0	0	0
HIFP	0	0	0	207813	207813	0	0	207813	207813	207813
PA	0	0	3643.9	4818.6	4818.6	0	0	4818.6	4818.6	0
$RhCl_3$	0	0	0	681.2	681.2	0	0	681.2	681.2	0
HI	0	0	0	3331.0	3331.0	0	0	3331.0	3331.0	3331.0
CO_2	0	0	0	2862.7	2862.7	0	0	2862.7	2862.7	0
TPA	0	0	0	10806.3	10806.3	10806.3	10806.3	0	0	0

The unit in the material list is kg/h.

Table S9-II. Continued.

Para.	CO2	LIQUID	LIQUID1	RH	PA	PA2	PA0	H2O	PA3	PA1
Temp./°C	60	60	60	60	162.3	33	118.6	118.6	118.6	33
Pres./bar	1.0	1.0	1.0	1.0	1.9	1.0	1.0	1.0	1.0	1.0
WATER	0	56.3	56.3	0.0008	0.0001	0.0001	56.3	56.3	0	0
CO	0	0	0	0	0	0	0	0	0	0
PET	0	0	0	0	0	0	0	0	0	0
HIFP	0	0	0	0	0	0	0	0	0	0
PA	0	4818.6	4818.5	0.07	3257.4	3257.4	1561.1	0	1561.	1561.1
$RhCl_3$	0	681.2	0	681.2	0	0	0	0	0	0
HI	0	0	0	0	0	0	0	0	0	0
CO_2	2862.7	0	0	0	0	0	0	0	0	0
TPA	0	0	0	0	0	0	0	0	0	0

The unit in the material list is kg/h.

6.4 Utility:

Table S10. Utility of PET carbonylolysis system.

Unit processes used	Type of utility	Quality	Function
REACTOR	LQS	241072.0 kg/h	Cooling reactor
COOLER1	KQ	241072.0 kg/h	Cool the product and co-product steam
COOLER3	KQ	1561.1 kg/h	Cooling
COOLER4	KQ	3257.4 kg/h	Cooling
PUMP	DIAN	10806.3 kg/h	Decompression
HEATER1	DYZQ	230265.0 kg/h	Heating
RADFAC	LQS	3257.4 kg/h	Cool the top fraction
	ZYZQ	1617.5 kg/h	Heat the tower kettle
SEP-1	DYZQ	241072.0 kg/h	Heating
SEP-2	DYZQ	230265.0 kg/h	Heating
SEP-3	DYZQ	1617.5 kg/h	Heating

Table S11. Details of the utilities of PET carbonylolysis system.

Type of utility used	Initial and final state of utility	
	Initial	Final
LQS	20 °C; 1 atm; liquid phase	25 °C; 1 atm; liquid phase
KQ	30 °C; 1 atm; liquid phase	35 °C; 1 atm; liquid phase
DIAN	-	-
DYZQ	125 °C; 2.32 bar; gaseous phase	124 °C; 2.25 bar; liquid phase
ZYZQ	175 °C; 8.91 bar; gaseous phase	174 °C; 8.70 bar; liquid phase

6.5 Energy balance:

Table S12-I. Energy balance of PET carbonylolysis system.

Equipment	REATOR	COOLER1	COOLER3	COOLER4	HEATER1
Heat duty/kw	-18373.9	-21931.5	-49.0	-167.8	14944.1

Table S12-II. Continued.

Equipment	RADFAC		SEP-1	SEP-2	SEP-3
Heat duty/kw	CON: -342.1	REB: 517.8	0	444.9	30.9

6.6 Goal and scope:

Table S13. Goal and scope of this LCA study.

Goal	
Reason and scope	Focus on carbon dioxide emissions and consumption of non-renewable energy.
Audience	Industrial stakeholders, the research community, and the public
Application	Provide technical support for polyester plastic carbon emission reduction policies and circular economy
Intention to use results in comparative studies	Yes, the results are to be compared and disclosed to the public through this article's publication
Scope	
Product system	The PET waste depolymerization is based on CN and EU.
Functional unit	1 kg of waste PET chips
System boundary[a]	Cradle-to-factory gate
Allocation	Waste PET cut-off, all environmental effects are allocated to TPA and PA
Assumptions	(I) The pre-treatment discharge of waste PET is consistent with the mechanical method<br>(II) This system deals with 100000 tons per year<br>(III) This system is located in CN or EU
Requirements on data and quality	Foreground material and energy consumption data were obtained from simulation in Aspen Plus V11 and the background processes were chosen based on Open LCA 2.4.0.
Impact categories assessed	1. GWP, 100a, kg CO_2 equivalent.<br>2. NREU, MJ.
limitations	In addition to the above-mentioned assumptions, the following aspects are not assessed: plant construction and equipment maintenance.
Report requirements	To present the outcome via journal publication which is openly accessible to everyone.

[a] The analysis adopted a cradle-to-gate system boundary with cut-off approach. The PET waste recycling technology boundary covered: (1) mechanical shredding of waste PET (collection, transportation, and pretreatment), (2) PA production, and (3) PET carbonylation depolymerization and separation.

6.7 NREU and GWP of each raw material:

Table S14. NREU values of each raw material of post-consumer PET chips carbonylolysis used in the LCA.

	NREU (CN)		NREU (EU)	
Subprocess	Value	Unit	Value	Unit
waste PET	3.65	MJ/Kg	3.48	MJ/Kg
H_2O	0.00326	MJ/Kg	0.00201	MJ/Kg
CO	16.1	MJ/Kg	13.3	MJ/Kg
Heat (MJ)	3.2	MJ/MJ	4.17	MJ/MJ
Total	22.95	MJ/Kg	20.95	MJ/Kg

Table S15. GWP values of each raw material of post-consumer PET chips carbonylolysis used in the LCA.

	GWP (CN)		GWP (EU)	
Subprocess	Value	Unit	Value	Unit
waste PET	0.288	Kg $CO_{2\text{-eq}}$/Kg	0.292	Kg $CO_{2\text{-eq}}$/Kg
H_2O	0.000293	Kg $CO_{2\text{-eq}}$/Kg	0.000175	Kg $CO_{2\text{-eq}}$/Kg
CO	0.645	Kg $CO_{2\text{-eq}}$/Kg	0.392	Kg $CO_{2\text{-eq}}$/Kg
CO_2	0.228	Kg $CO_{2\text{-eq}}$/Kg	0.228	Kg $CO_{2\text{-eq}}$/Kg
Heat (MJ)	0.258	Kg $CO_{2\text{-eq}}$/Kg	0.257	Kg $CO_{2\text{-eq}}$/Kg
Total	1.42	Kg $CO_{2\text{-eq}}$/Kg	1.17	Kg $CO_{2\text{-eq}}$/Kg

6.8 Techno-economic analysis:

Table S16. Techno-economic analysis of PET recycling.

Items		Quantity	Unit
Plant costs	Capital cost	12.96	million USD per year
	Equipment cost	0.39	million USD per year
	Installed cost	1.68	million USD per year
	Total	15.03	million USD per year
Working costs	Operating cost	2.13	million USD per year
	Utilities cost	0.97	million USD per year
	Total	3.10	million USD per year
Total raw materials cost	Waste PET	44.45	million USD per year
	CO	10.96	million USD per year
	Total	55.41	million USD per year
Total production sales	TPA	66.62	million USD per year
	Propionic Acid	42.84	million USD per year
	Total	109.46	million USD per year
Annual profit		35.92	million USD per year

[a]Material loss means the total loss of catalyst, solvent and catalytic additives that need to be replenished during the actual production process.

Supplementary References

1. Fuanin RU, Junia BDF, Shinguruton TK. Rhodium catalyst regeneration method Patent JPS581974B.
2. Thomas CM, Süss-Fink G. Ligand effects in the rhodium-catalyzed carbonylation of methanol. *Coord Chem Rev* **243**, 125–142 (2003).
3. Smith B, Torrence G, Murphy M, Aguilo A. The rhodium-catalyzed methanol carbonylation to acetic acid at low water concentrations: the effect of iodide and acetate on catalyst activity and stability. *J Mol Catal* **39**, 115–136 (1987).
4. Haynes A. Catalytic methanol carbonylation. In: *Advances in catalysis*. Elsevier (2010).
5. Shang W, Gao M, Chai Y, Wu G, Guan N, Li L. Stabilizing isolated rhodium cations by MFI zeolite for heterogeneous methanol carbonylation. *ACS Catal* **11**, 7249–7256 (2021).
6. Yang C-C, Kilos BA, Barton DG, Weitz E, Notestein JM. The role of iodide promoters and the mechanism of ethylene carbonylation catalyzed by molybdenum hexacarbonyl. *J Catal* **319**, 211–219 (2014).
7. Yacob S, Park S, Kilos BA, Barton DG, Notestein JM. Vapor-phase ethanol carbonylation with heteropolyacid-supported Rh. *J Catal* **325**, 1–8 (2015).
8. Mei Q*, et al.* Selective utilization of the methoxy group in lignin to produce acetic acid. *Angew Chem Int Ed* **56**, 14868–14872 (2017).
